# Supplementary material for: Discovery and application of insertion-deletion (INDEL) polymorphisms for QTL mapping of early life-history traits in Atlantic salmon
Source: BMC Genomics. 2010 Mar 8;11:156. doi: 10.1186/1471-2164-11-156 (PMC2838853; doi:10.1186/1471-2164-11-156)
Supplement: Additional file 2 — Information on developed 76 locus single-run INDEL panel in Atlantic salmon. Information on fluorescence labeling, primer concentrations, PCR pooling and links to alignments, INDEL motifs and GENESCAN (Burge and Karlin 1997) predictions of genes/exons are available in html format. [file 1471-2164-11-156-S2.ZIP › Additionalfile2/snpsummary8396.html]

```
Cluster 2619 Contig 1

prev  Summary    Contig List  next
```

Size of Consensus sequence = 1067

Number of sequences = 21

Minimum redundancy = 6

Key

A gi|117471556|gb|EG803775.1|EG803775 EST\_ssal\_evd\_916 ssalevd thymus Salmo salar cDNA Salmo salar cDNA clone ssal\_evd\_005\_188\_rev 5', mRNA sequence  
B gi|117472871|gb|EG805090.1|EG805090 EST\_ssal\_evd\_5900 ssalevd thymus Salmo salar cDNA Salmo salar cDNA clone ssal\_evd\_506\_222\_rev 5', mRNA sequence  
C gi|117442019|gb|EG774242.1|EG774242 EST\_ssal\_evd\_49602 ssalevd thymus Salmo salar cDNA Salmo salar cDNA clone ssal\_evd\_566\_283\_rev 5', mRNA sequence  
D gi|117475642|gb|EG807861.1|EG807861 EST\_ssal\_evd\_28395 ssalevd thymus Salmo salar cDNA Salmo salar cDNA clone ssal\_evd\_537\_064\_rev 5', mRNA sequence  
E gi|117447281|gb|EG779500.1|EG779500 EST\_ssal\_evd\_36334 ssalevd thymus Salmo salar cDNA Salmo salar cDNA clone ssal\_evd\_548\_192\_rev 5', mRNA sequence  
F gi|117454877|gb|EG787096.1|EG787096 EST\_ssal\_evd\_7707 ssalevd thymus Salmo salar cDNA Salmo salar cDNA clone ssal\_evd\_509\_008\_rev 5', mRNA sequence  
G gi|117450664|gb|EG782883.1|EG782883 EST\_ssal\_evd\_39378 ssalevd thymus Salmo salar cDNA Salmo salar cDNA clone ssal\_evd\_552\_269\_rev 5', mRNA sequence  
H gi|117451967|gb|EG784186.1|EG784186 EST\_ssal\_evd\_40552 ssalevd thymus Salmo salar cDNA Salmo salar cDNA clone ssal\_evd\_554\_119\_rev 5', mRNA sequence  
I gi|117438365|gb|EG770588.1|EG770588 EST\_ssal\_evd\_46313 ssalevd thymus Salmo salar cDNA Salmo salar cDNA clone ssal\_evd\_562\_083\_rev 5', mRNA sequence  
J gi|117464497|gb|EG796716.1|EG796716 EST\_ssal\_evd\_16365 ssalevd thymus Salmo salar cDNA Salmo salar cDNA clone ssal\_evd\_520\_334\_rev 5', mRNA sequence  
K gi|117438366|gb|EG770589.1|EG770589 EST\_ssal\_evd\_46314 ssalevd thymus Salmo salar cDNA Salmo salar cDNA clone ssal\_evd\_562\_083\_fwd 3', mRNA sequence  
L gi|117461082|gb|EG793301.1|EG793301 EST\_ssal\_evd\_13291 ssalevd thymus Salmo salar cDNA Salmo salar cDNA clone ssal\_evd\_516\_250\_rev 5', mRNA sequence  
M gi|117460451|gb|EG792670.1|EG792670 EST\_ssal\_evd\_12722 ssalevd thymus Salmo salar cDNA Salmo salar cDNA clone ssal\_evd\_515\_339\_rev 5', mRNA sequence  
N gi|117442020|gb|EG774243.1|EG774243 EST\_ssal\_evd\_49603 ssalevd thymus Salmo salar cDNA Salmo salar cDNA clone ssal\_evd\_566\_283\_fwd 3', mRNA sequence  
O gi|117461081|gb|EG793300.1|EG793300 EST\_ssal\_evd\_13290 ssalevd thymus Salmo salar cDNA Salmo salar cDNA clone ssal\_evd\_516\_250\_fwd 3', mRNA sequence  
P gi|117454878|gb|EG787097.1|EG787097 EST\_ssal\_evd\_7708 ssalevd thymus Salmo salar cDNA Salmo salar cDNA clone ssal\_evd\_509\_008\_fwd 3', mRNA sequence  
Q gi|117451968|gb|EG784187.1|EG784187 EST\_ssal\_evd\_40553 ssalevd thymus Salmo salar cDNA Salmo salar cDNA clone ssal\_evd\_554\_119\_fwd 3', mRNA sequence  
R gi|117447280|gb|EG779499.1|EG779499 EST\_ssal\_evd\_36333 ssalevd thymus Salmo salar cDNA Salmo salar cDNA clone ssal\_evd\_548\_192\_fwd 3', mRNA sequence  
S gi|117460450|gb|EG792669.1|EG792669 EST\_ssal\_evd\_12721 ssalevd thymus Salmo salar cDNA Salmo salar cDNA clone ssal\_evd\_515\_339\_fwd 3', mRNA sequence  
T gi|117475641|gb|EG807860.1|EG807860 EST\_ssal\_evd\_28394 ssalevd thymus Salmo salar cDNA Salmo salar cDNA clone ssal\_evd\_537\_064\_fwd 3', mRNA sequence  
U gi|117450665|gb|EG782884.1|EG782884 EST\_ssal\_evd\_39379 ssalevd thymus Salmo salar cDNA Salmo salar cDNA clone ssal\_evd\_552\_269\_fwd 3', mRNA sequence

5 SNPs detected

A B C D E F G H I J K L M N O P Q R S T U  cosegregation weighted

79 - - - A A A A A - - - - A . - A . A A . .   4/5 64.76
80 - - - A A A A A - - - - A . - A . A A . .   4/5 64.76
81 - - - T T T T T - - - - T . - T . T T . .   4/5 64.76
82 - - - T T T T T - - - - T . - T . T T . .   4/5 64.76
274 . C C A A A A A C C C A A C A A A A A A A   1/5 19.05
